# Software-assisted structured reporting and semi-automated TNM classification for NSCLC staging in a multicenter proof of concept study

**DOI:** 10.1186/s13244-024-01836-z

**Published:** 2024-10-28

**Authors:** Maurice M. Heimer, Yevgeniy Dikhtyar, Boj F. Hoppe, Felix L. Herr, Anna Theresa Stüber, Tanja Burkard, Emma Zöller, Matthias P. Fabritius, Lena Unterrainer, Lisa Adams, Annette Thurner, David Kaufmann, Timo Trzaska, Markus Kopp, Okka Hamer, Katharina Maurer, Inka Ristow, Matthias S. May, Amanda Tufman, Judith Spiro, Matthias Brendel, Michael Ingrisch, Jens Ricke, Clemens C. Cyran

**Affiliations:** 1 Department of Radiology, LMU University Hospital, LMU Munich, Munich, Germany; 2 Bavarian Cancer Research Center (BZKF), Erlangen, Germany; 3 Department of Statistics, LMU Munich, Munich, Germany; 4 Munich Center for Machine Learning (MCML), Munich, Germany; 5 Department of Nuclear Medicine, LMU University Hospital, LMU Munich, Munich, Germany; 6 Department of Diagnostic and Interventional Radiology, Technical University of Munich, Munich, Germany; 7 Department of Diagnostic and Interventional Radiology, University Hospital Würzburg, Würzburg, Germany; 8 Department of Diagnostic and Interventional Radiology and Neuroradiology, University Hospital Augsburg, Augsburg, Germany; 9 Institute of Radiology, University Hospital Erlangen, Erlangen, Germany; 10 Department of Radiology, University Hospital Regensburg, Regensburg, Germany; 11 Department of Diagnostic and Interventional Radiology and Nuclear Medicine, University Medical Center Hamburg Eppendorf, Hamburg, Germany; 12 Department of Pneumology, LMU University Hospital, LMU Munich, Munich, Germany; 13 Comprehensive Pneumology Center (CPC-M), Member of the German Center for Lung Research (DZL), Munich, Germany; 14 German Center for Neurodegenerative Diseases (DZNE), Munich, Germany; 15 Munich Cluster for Systems Neurology (SyNergy), Munich, Germany

**Keywords:** Lung, Non-small-cell lung carcinoma, PET-CT, TNM classification

## Abstract

**Objectives:**

In this multi-center study, we proposed a structured reporting (SR) framework for non-small cell lung cancer (NSCLC) and developed a software-assisted tool to automatically translate image-based findings and annotations into TNM classifications. The aim of this study was to validate the software-assisted SR tool for NSCLC, assess its potential clinical impact in a proof-of-concept study, and evaluate current reporting standards in participating institutions.

**Methods:**

A framework for SR and staging of NSCLC was developed in a multi-center collaboration. SR annotations and descriptions were used to generate semi-automated TNM classification. The SR and TNM classification tools were evaluated by nine radiologists on *n* = 20 representative [18F]FDG PET/CT studies and compared to the free text reporting (FTR) strategy. Results were compared to a multidisciplinary team reference using a generalized linear mixed model (GLMM). Additionally, participants were surveyed on their experience with SR and TNM classification.

**Results:**

Overall, GLMM analysis revealed that readers using SR were 1.707 (CI: 1.137–2.585) times more likely to correctly classify TNM status compared to FTR strategy (*p* = 0.01) resulting in increased overall TNM correctness in 71.9% (128/178) of cases compared to 62.8% (113/180) FTR. The primary source of variation in classification accuracy was explained by case complexity. Participants rated the potential impact of SR and semi-automated TNM classification as positive across all categories with improved scores after template validation.

**Conclusion:**

This multi-center study yielded an effective software-assisted SR framework for NSCLC. The SR and semi-automated classification tool improved TNM classification and were perceived as valuable.

**Critical relevance statement:**

Software-assisted SR provides robust input for semi-automated rule-based TNM classification in non-small-cell lung carcinoma (NSCLC), improves TNM correctness compared to FTR, and was perceived as valuable by radiology physicians.

**Key Points:**

- SR and TNM classification are underutilized across participating centers for NSCLC staging.
- Software-assisted SR has emerged as a promising strategy for oncologic assessment.
- Software-assisted SR facilitates semi-automated TNM classification with improved staging accuracy compared to free-text reports in NSCLC.

**Graphical Abstract:**

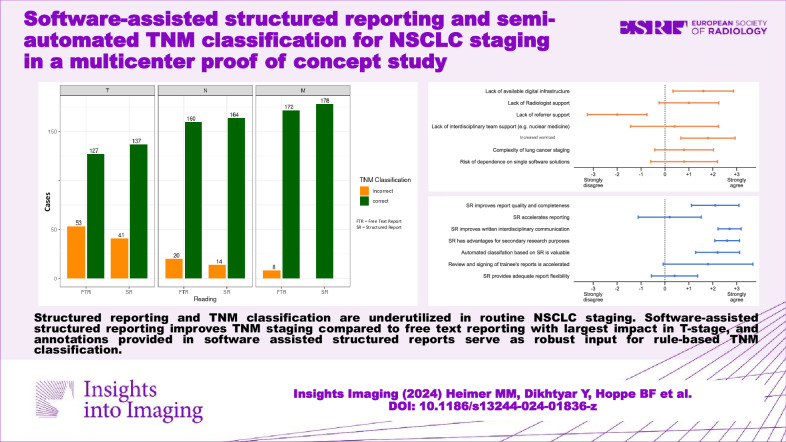

## Introduction

Recent studies have demonstrated that structured reporting (SR) contributes to improved report completeness and precision across various cancer entities and helps to close the communication quality gap [1–8]. However, widespread adoption of SR into clinical routine remains far from reality, due to a lack of monetary and structural incentives, the absence of technical standards, and radiologists’ habituation towards prose-free text reporting (FTR). Expanding on the framework of report standardization, Nobel et al have proposed that software-assisted solutions present an indispensable prerequisite to facilitate SR [9, 10]. Both the European Society of Radiology (ESR) and the Radiological Society of North America (RSNA) advocate SR as a key element in advancing value-based radiology, leveraging data for secondary use and research, enabling quality assurance initiatives, and reducing structural mistakes [9, 11].

Lung cancer is a major global health burden, with an estimated annual incidence of 2 million new diagnoses and 1.76 million deaths per year. Non-small cell lung cancer (NSCLC) accounts for 85–90% of all lung cancer cases [12, 13]. Imaging plays a central role in the detection, staging, and post-treatment surveillance of NSCLC making it a routine diagnostic task in oncologic and thoracic radiology practice [14, 15]. Detailed information on lung cancer staging of the primary tumor (T-category), regional lymph node involvement (N-category), and distant metastasis (M-category) is provided by the 8th edition of the TNM criteria. However, TNM classification is rarely explicitly and clearly included in clinical radiology reports [16, 17].

The aim of this multicenter pilot study was to assess the effectiveness of a collaboratively developed SR framework featuring semi-automated TNM classification of NSCLC within a CE-certified digital platform. The study evaluated institutional standards, preferences, and perceptions of clinical radiologists regarding SR of NSCLC and compared SR and FTR strategies for TNM classification.

## Methods

### Ethics statement

This retrospective study was approved by the institutional ethics committee (approval number 22-0416). Informed consent was waived.

### Development of a structured report template

The Bavarian Oncologic Radiology Network (BORN—https://bzkf.de/born/?lang=en) has evolved as a regional platform to facilitate multi-institutional imaging protocol and reporting harmonization for a variety of tumor entities including NSCLC. Participating centers include radiology departments from LMU Hospital Munich, Technical University Munich, University Hospital of Erlangen, University Hospital of Regensburg, University Hospital of Würzburg, and University Hospital of Augsburg. Appointed thoracic radiology experts of all respective university hospitals devised an oncological framework for SR of NSCLC based on the 8th edition of the TNM classification and was established through consensus, following a process analogous to the Delphi method [17]. TNM descriptors were itemized and structured hierarchically using an image-based software tool built in Mint Lesion^TM^ (Mint Medical GmbH, Heidelberg, Germany). The detailed template script is found online (https://bzkf.de/born-template-lungenkarzinom/). Additionally, the SR tool was enriched with a semi-automated, rule-based engine designed to translate SR annotations and descriptions into TNM classification.

### Survey design

Physician survey participants (*n* = 10) were selected by their respective institutions, representing all contributing centers. The questionnaire included demographic questions including professional experience and previous exposure to lung cancer imaging. Additionally, the questionnaire assessed the comprehensiveness of radiology reports in the participants’ institutions, as well as preferences and expectations concerning SR implementation, questionnaire items are shown in Table 1. Each item was evaluated using a 7-point Likert scale, with responses ranging from −3 (strongly disagree) over 0 to +3 (strongly agree).

**Table 1:** Questionnaire items are grouped by task into demographic (D), SR general (SR-G), and SR barriers (SR-B) questions that were assessed once, as well as further questions on SR that were assessed before and after the validation task (SR-PP)

Item number	Task	Question—item	Metric
1	D	How many years of radiology experience do you have?	Years
2	D	How many NSCLC stagings do you estimate to have been reported?	Arbitrary
3	D	Is SR used in your department for NSCLC reporting?	Yes/no
4	D	Has SR been used in any form in your department?	Yes/no
5	D	In how many cases is TNM reported for NSCLC in your department?	%
6	SR-G	Would you generally use SR in practice?	7 Likert scale
7	SR-G	SR improves report quality and completeness?	7 Likert scale
8	SR-G	SR accelerates reporting?	7 Likert scale
9	SR-G	Should SR software be integrated into PACS?	7 Likert Scale
10	SR-G	SR improves written interdisciplinary communication?	7 Likert scale
11	SR-G	SR has advantages for secondary research purposes?	7 Likert scale
12	SR-G	Automated classification based on SR is valuable?	7 Likert scale
13	SR-G	Review and signing of trainee’s reports is accelerated?	7 Likert scale
14	SR-G	SR provides adequate report flexibility?	7 Likert scale
15	SR-B	Lack of available digital infrastructure?	7 Likert scale
16	SR-B	Lack of radiologist support?	7 Likert scale
17	SR-B	Lack of referrer support?	7 Likert scale
18	SR-B	Lack of interdisciplinary team support (e.g., nuclear medicine)?	7 Likert scale
19	SR-B	Does SR increase workload?	7 Likert scale
20	SR-B	Is TNM for NSCLC too complex for SR?	7 Likert scale
21	SR-B	Is there a risk of dependence on single software solutions?	7 Likert scale
22	SR-PP	Do you know the TNM criteria for NSCLC staging adequately?	7 Likert scale
23	SR-PP	Are you confident in providing TNM without support tools?	7 Likert scale
24	SR-PP	Would you trust your annotations to be adequate for automated classification tasks?	7 Likert scale
25	SR-PP	Would you trust semi-automated TNM classification based on your annotation to be more accurate than your own unassisted TNM?	7 Likert scale
26	SR-PP	Structured annotation increases awareness of TNM criteria?	7 Likert scale
27	SR-PP	Image-guided annotation assistance improves classification for N-stage?	7 Likert scale
28	SR-PP	SR improves T-staging?	7 Likert scale
29	SR-PP	SR improves N-staging?	7 Likert scale
30	SR-PP	SR improves M-staging?	7 Likert scale
31	SR-PP	SR improves local/curative stage NSCLC?	7 Likert scale
32	SR-PP	SR improves advanced/palliative stage NSCLC?	7 Likert scale
33	SR-PP	SR improves all stage NSCLC?	7 Likert scale
34	SR-PP	SR improves staging NSCLC at diagnosis?	7 Likert scale
35	SR-PP	SR improves response assessment?	7 Likert scale
36	SR-PP	SR improves surveillance?	7 Likert scale

Items are numbered consecutively. Metrics used to evaluate the individual items are shown in the right column

*D* demographic, *SR* structured reporting, *SR-G* structured reporting general, *SR-B* structured reporting barriers, *SR-PP* structured reporting pre-post

### Validation of the structured report template

A total of nine physician participants from five of the six participating institutions attended a supervised in-person evaluation workshop and received comprehensive training to use the SR template. Each attendee independently reviewed *n* = 20 representatives portal-venous phase contrast-enhanced 18F-Fluorodeoxyglucose ([18F]FDG) PET/CT studies selected at a single center to represent a diverse cohort of NSCLC patients (Table 2). Image findings were annotated and characterized using the SR template. For the TNM assessment, participants received pictorial-based guidance based on multidisciplinary team (MDT) meeting decisions that served as reference standards. To mitigate bias, participants were blinded to TNM outcomes generated by the semi-automated classification tool. Also, participants received access to the 8th edition TNM classification NSCLC manual. Before and after the evaluation task, participants were asked to rate a series of questions regarding their perception of the potential impact of a structured report and an automated TNM classification tool on their individual routine in analogy to the above-mentioned survey.

**Table 2:** Demographics of representative patients selected for the [18F]FDG PET/CT validation task

Variable		Value	
Age		68 ± 10.5	years
Sex	Male	9/20 (45%)	
	Female	11/20 (55%)	
BMI		25.1 ± 4.2	kg/m^2^
Smoking history	(yes)	14/20 (70%)	
Pack years		30.8 ± 26.7	years
Previous lung disease		6/20 (30%)	
Previous cancer disease		5/20 (25%)	
Histology	Adenocarcinoma	12/20 (60%)	
	Squamous cell carcinoma	7/20 (35%)	
	Neuroendocrine carcinoma	1/20 (5%)	
UICC-stage	I	4/20 (20%)	
	II	3/20 (15%)	
	III	6/20 (30%)	
	IV	7/20 (35%)	
Hemoglobin		13.5 ± 1.5	g/dL
C-reactive Protein (CRP)		9.3 ± 15.2	mg/dL

Data are shown as counts (*n*) including ± standard deviation where appropriate

### Assessment of the validation task

The TNM output of the rule-based semi-automated SR classification was assessed for correctness when compared to input. Semi-automated SR and FTR TNM classifications were compared to the MDT reference. Discrepancies were assessed with regard to individual T-, N-, and M classifiers, as well as aggregate TNM classification and whether errors lead to upstaging or downstaging. Reasons for deviation were analyzed using the SR annotations.

### Statistics

To address the dependence structure arising from repeated measurements, we employed a generalized linear mixed model (GLMM), specifically a logistic regression model, for binary classification. This model was utilized to evaluate the effects of the reading method and TNM category on correct and incorrect classifications. The GLMM also accommodates the intraclass correlation coefficient (ICC) between observations made by the same reader on multiple images to test for differences between SR, as well as FTR and to assess variations in the classification of TNM categories, while properly accounting for the intra-reader dependencies [18]. Additionally, post-hoc pairwise comparisons of the effects on TNM were performed via the Sidak correction to control for multiple comparisons. Analyses were performed in R (version 4.3.2). The survey results were analyzed using the non-parametric Wilcoxon signed rank test for matched-pair comparison. Fleiss Kappa was used to test inter-reader reliability. The correlation between experience and occurrence of TNM errors was analyzed using Spearman correlation. Two-sided significance testing was conducted with an α of 5% (*p* < 0.05). All tests used for survey analysis were performed using IBM SPSS Statistics (Version 29, IBM Corp. IBM SPSS Statistics for Windows, Armonk, NY). The results of the questionnaires were visualized using Python (version 3.10.9) with recent SciPy and Seaborn libraries.

## Results

### Participant characteristics and use of SR

A total of ten radiologists from all six participating institutions completed the demographic and exploratory survey on SR, with 3/10 (30%) female responders. These physicians had various levels of radiology experience (mean 6.3 years; range 2–12 years), with self-estimated previous 423 ± 351 reported lung cancer staging examinations per reader. The survey revealed that none of the institutions (0/6) had implemented SR in clinical routine lung cancer staging prior to the study, with participants from two institutions (2/6) reporting previous exposure to SR in lung cancer. Across institutions, participants responded that to their perception TNM classification is reported infrequently in approximately 9.2% (range 0–30%) of clinical reports.

### Survey on structured reporting (SR)

**Fig. 1:**
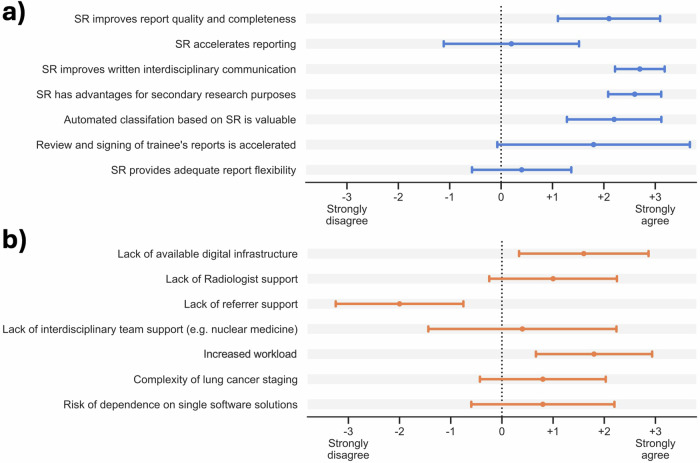
Participants' opinion on SR and perceived barriers of clinical implementation. The perception of physicians to general statements regarding SR and potential barriers regarding SR are shown in (**a**, **b**), respectively. The survey reflects an overall positive perception regarding SR and perspectives on its clinical implementation. Participants rated a lack of digital infrastructure and perceived increased reporting time as the most relevant obstacles to clinical translation. SR, structured reporting

Overall, participating physicians revealed their general positive perception towards SR for NSCLC, scoring 2.3 ± 0.5 on the question “Would you generally use SR in practice” as shown in Fig. 1a. Reasons, why SR is not implemented in clinical routine, are summarized in Fig. 1b. The most relevant arguments scored in the survey why SR is currently not implemented in clinical practice were perceived increased workload (1.8 ± 1.2), and lack of digital infrastructure or software (1.6 ± 1.4).

### Validation of the structured report template

In total, a cohort of *n* = 9 radiologists (experience means 5.7 years; range 2–10 years) participated in the on-premises validation task, including representatives from five of the six institutions. Participants completed all readings based on the simulated MDT, providing semi-automated SR-assisted (*n* = 178) and unassisted FTR (*n* = 180) TNM classification in a total of *n* = 20 representative [18F]FDG PET/CT NSCLC studies; *n* = 2 SR-classification cases of a single participant were not documented and could not be retrieved and were excluded.

**Fig. 2:**
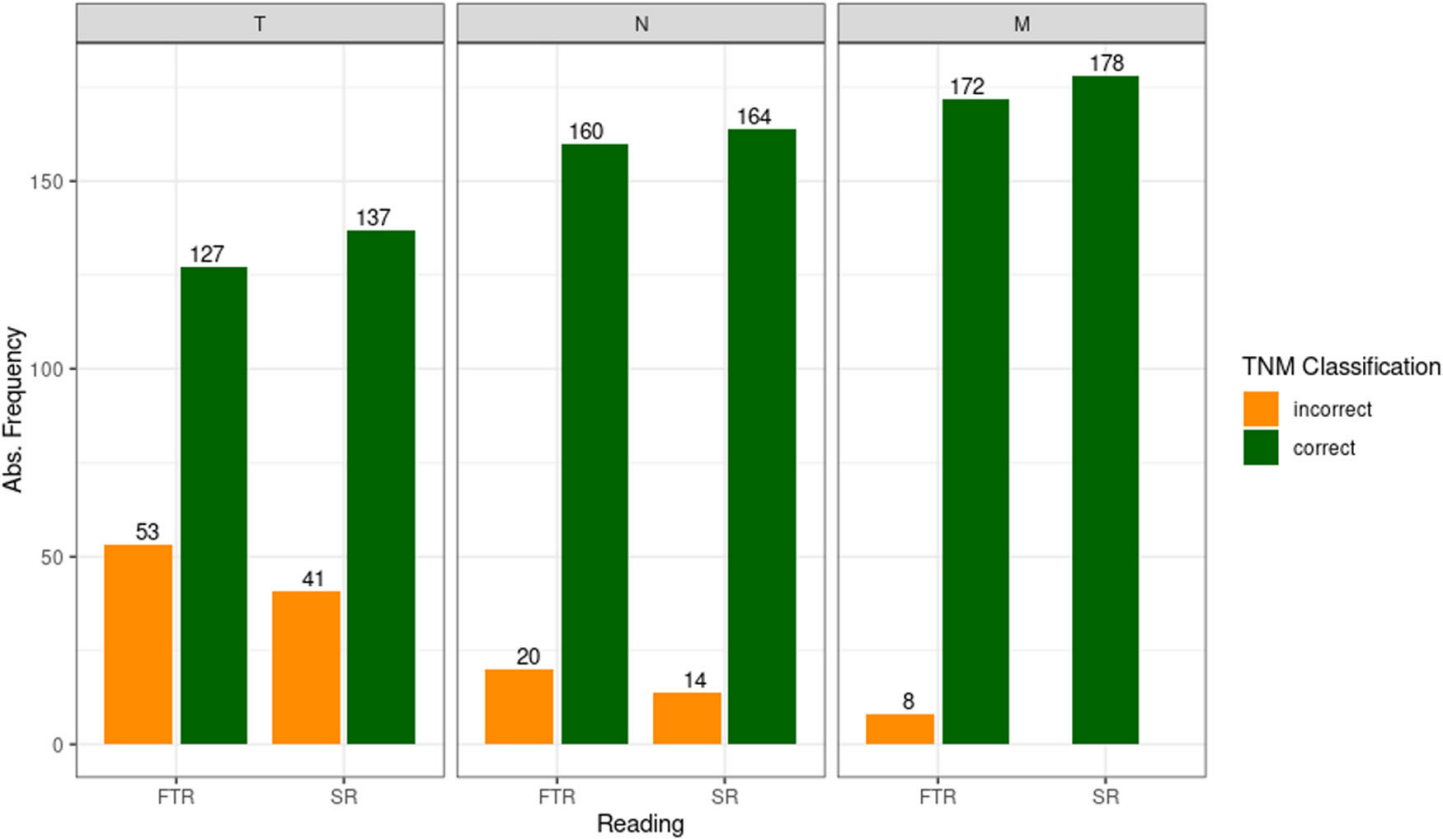
Classification performance (orange = incorrect and green = correct) of study participants with regard to individual TNM-descriptors, demonstrating improved accuracy of SR strategy across all categories compared to FTR. SR, structured reporting; FTR, free text reporting

**Fig. 3:**
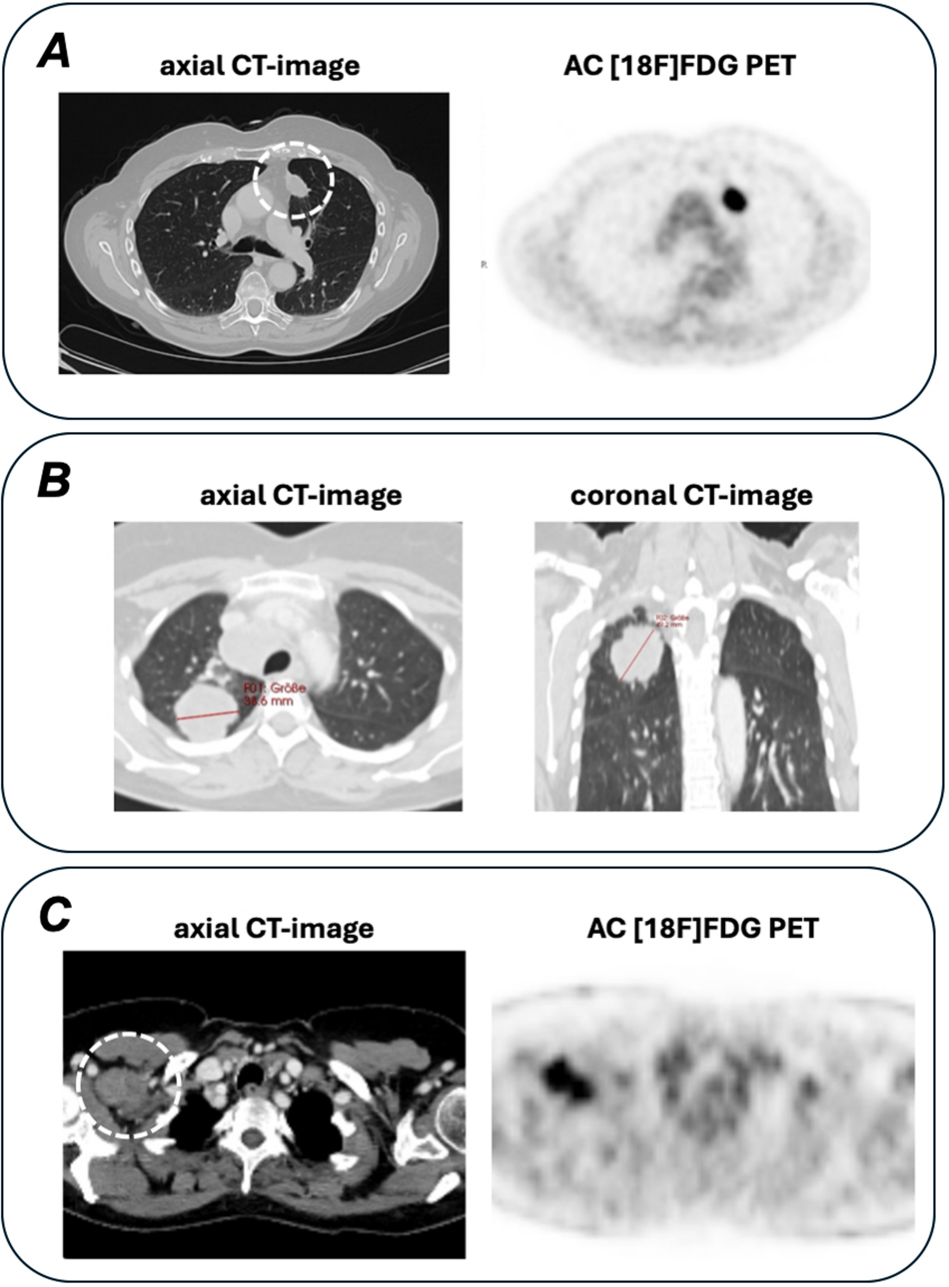
Representative classification errors in SR and FTR. **A** Shows the primary tumor in patient 3 with broad visceral pleural contact indicative of an infiltration of the visceral pleura (T2a), as compared to size-based stage T1c. **B** Shows the primary tumor in patient 13 demonstrating a maximum multiplanar diameter in the coronal plane of 4.9 cm (T2b) as compared to the maximum axial diameter of 3.9 cm (T2a). **C** Shows a right-sided axillary lymph node metastasis consistent with an extrathoracic metastasis instead of a regional lymph node (N3) as it is not included in the International Association for the Study of Lung Cancer (IASLC) map. SR, structured reporting; FTR, free text reporting; AC, attenuation correction; FDG, fluorodeoxyglucose

The semi-automated rule-based TNM classification provided correct output in all (178/178) cases with regard to participants' input. Overall, the use of semi-automated SR classification significantly (*p* = 0.01) increased overall TNM correctness in 71.9% (128/178) of cases compared to 62.8% (113/180) cases documented in FTR, as shown in Fig. 2. The majority of classification errors were observed in T-stage 25.7% (92/358), followed by N-stage 10.3% (37/358), and M-stage 2.2% (8/358). Semi-automated SR classification was superior to unassisted classification for T- (137 vs 127), N-stage (164 vs 160), and M-stage (178 vs 172) as shown in Fig. 2. Interpretation errors in SR and FTR classification resulted in aggregate TNM upstaging in 34 and 38 cases, as well as downstaging in 18 and 21 cases, respectively. In the FTR cohort, incomplete or inadequate TNM documentation (e.g., T2 - lacking the subcategory, or M2 - nonexistent) was found in eight cases, none in the SR cohort. Overall, in both SR and FTR classification errors were explained by inaccurate tumor size measurement (T-category; *n* = 43/358; 12.0%) and/or error in the description of local infiltration (T-category, e.g., local infiltration; *n* = 63/358; 17.6%), as well as mistakes in the assignment of anatomical location of lesions, regardless of individual TNM classifier (*n* = 48). Representative cases are displayed in Fig. 3. Intra-reader discrepancies between semi-automated SR and unassisted FTR TNM classification were observed in *n* = 55 (range 1–10) cases, regardless of classifier.

The GLMM revealed that there was a significant difference (*p* = 0.01) in overall TNM correctness between SR and FTR, with readers using SR having a 1.707 (CI: 1.137–2.585) times higher chance of correctly classifying TNM status compared to those using FTR (Table 3). The assumptions of the GLMM were verified and met. However, due to singularity issues, we were unable to consider a potential interaction between the reading method and the TNM classification. Instead, we included their fixed effects as separate predictors in the GLMM, along with random effects for both the readers and the images. The ICC of individual image studies was 0.278, indicating that approximately 27.8% of the total variance in classification accuracy is attributable to differences between image studies, compared to approximately 0.5% attributable to different readers. Overall, inter-reader agreement was very high (Kappa = 0.889, *p* < 0.001). There was no relevant correlation between clinical experience and classification errors in FTR ($\rho$ = 0.21; *p* = 0.96) and SR (*ρ* = −0.142; *p* = 0.71).

**Table 3:** Odds ratio (OR) for each fixed effect, along with upper and lower confidence interval (CI) boundaries (95%) are shown in (a)

**(a) Predictor**	**OR**	**2.5% CI**	**97.5% CI**	***p*-value**
Intercept	56.926	–	–	–
Method SR	1.707	1.137	2.585	0.010
Category N	0.203	0.085	0.430	< 0.001
Category T	0.051	0.022	0.102	< 0.001
**(b) Predictor**	**OR**	**2.2% CI**	**97.5% CI**	***p*-value**
M vs N	1.603	0.656	2.540	< 0.001
M vs T	2.981	2.087	3.880	< 0.001
N vs T	1.394	0.851	1.921	< 0.001
**(c) Random effects**	**Variance**	**Std. dev**.	**Groupwise ICC**	
Patient/image	1.273	1.128	0.278	
Reader	0.023	0.153	0.005	

The odds ratio (OR) for each fixed effect, along with upper and lower confidence interval (CI) boundaries (95%) are shown in (a). The reference category for the predictor method is FTR, for TNM is category M. Pairwise comparisons (odds ratio differences) for classification correctness between TNM stages, including 95% CI and *p*-values are shown in (b). Random effects analysis of the GLMM. Variance, standard deviation, and GroupWise ICC for patients/images and readers are shown in (c)

*FTR* free text reporting, *SR* structured reporting, *CI* confidence interval, *GLMM* generalized linear mixed model

**Fig. 4:**
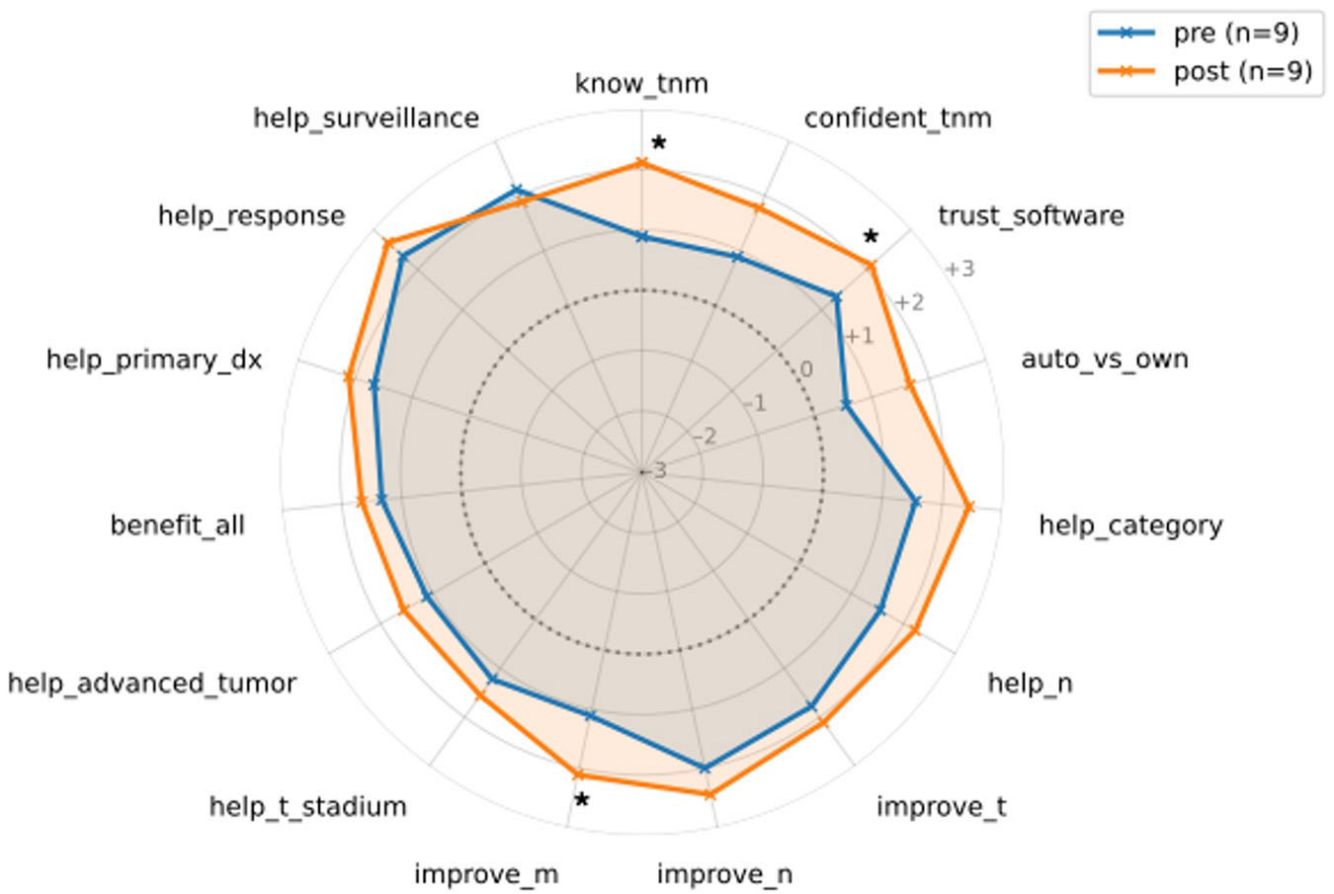
Spider plot visualizing the differences in assessment of SR (*n* = 9), plotting was based on the 7-point Likert scale responses (ranging from −3, “strongly disagree,” to +3, “strongly agree”). Questions 22–36 provided in Table 1 are presented in clockwise order. Overall, SR was perceived as a valuable reporting strategy across all categories by participants. Significantly improved perceptions are highlighted with an asterisk (*), demonstrating superior performance of the evaluated SR and classification tool. SR, structured reporting

The pre- and post-validation survey revealed an overall positive attitude towards SR across categories with increased preference after the validation task with regard to the following statements: “My understanding of TNM-staging increased using the SR” (*p* = 0.04), “M-staging is improved using SR” (*p* = 0.04) and “I trust the semi-automated TNM classification based on my annotations” (*p* = 0.04). Detailed findings are shown in Fig. 4.

## Discussion

In this multicenter study, we assessed institutional standards, preferences, and perceptions toward SR in NSCLC. A software-assisted SR tool for NSCLC featuring semi-automated TNM classification was developed in multicenter consensus for report harmonization. Performance was evaluated on a representative [18F]FDG PET/CT cohort of patients and compared to the conventional FTR strategy.

### Survey on SR

The survey revealed that SR had not been adopted for NSCLC reporting at any of the participating institutions, with TNM classification being underutilized and reported in less than 10% (0–30%) of clinical reports at staging. Only two of ten participants (20%) reported previous experience using SR for NSCLC. While SR has evolved as a dominant strategy for clinical trial reporting and imaging biomarker research it has still not been widely adopted in clinical routine [6, 19]. A recent international survey by the European Society of Oncologic Imaging including 200 radiologists from 51 countries revealed that 37.5% of radiologists who already used SR in clinical practice (*n* = 114) utilized SR for lung cancer reporting [6].

In this study, SR was accepted widely among radiologists for NSCLC across all examined categories, regardless of experience. Our findings indicate that radiologists perceive SR as a valuable strategy for NSCLC staging which is in line with previously reported experiences in oncologic imaging [20, 21]. Report standardization ensures that essential information is included uniformly in radiology reports and facilitates adherence to established guidelines and protocols, ensuring compliance with regulatory requirements and accreditation standards. Also, SR has the potential to reduce ambiguity and ultimately close the communication gap between healthcare professionals [22–24]. These arguments are reinforced by strategic perspectives outlined by the ESR and the RSNA aimed at enhancing value-based radiology [9, 11].

The survey revealed that perceived increased workload and reporting time, as well as, lack of digital infrastructure for software-assisted SR were perceived as the most relevant obstacles for routine clinical implementation. While increased reporting time likely has to be anticipated with single time point assessment at staging, SR may evolve as a valuable investment for longitudinal response assessment [25, 26]. Ristow et al found that software-assisted SR improved immune response criteria in solid tumor (iRECIST) assessment with reduced reporting time, reduced error, and higher inter-reader agreement compared to a manual approach [25]. Also, the survey suggests that inter-operability between software-assisted SR frameworks and local reporting and picture archiving and communication system (PACS) environment is important to consider, reducing workflow-related friction between different applications and software. Additionally, SR has been discussed as an essential tool for lung cancer screening programs and may have a significant positive impact on the training environment and reduce proofreading time to finalize and sign off trainee reports [8, 22, 23, 27–29].

### Validation of the SR template

Our study demonstrated that image-based annotations provided by software-assisted SR serve as robust input for semi-automated rule-based NSCLC TNM classification. The SR classification tool assigned correct TNM output in all cases with regard to input. However, interpretation errors affected SR and FTR equally. Overall, SR classification outperformed unassisted FTR classification and improved objective confirmability, which is explained by improved adherence to TNM criteria and more precise lesion annotation. The GLMM analysis revealed that the primary source of variation in classification accuracy was due to single complex studies rather than inter-reader variability. T-stage was misclassified for primarily two reasons including incorrect size measurement and assessment of local infiltration, while N- and M-stage classification errors were due to incorrect lesion localization. Readers using SR were significantly more likely (*p* = 0.01) to correctly classify TNM status compared to FTR, taking case complexity and reader variability into account. Improved TNM correctness resulted in both reduced TNM up- and downstaging. Errors carried forward from both SR and FTR may be translated into erroneous TNM classification which potentially has critical implications for patients if not reassessed and corrected in MDT [16].

With the growing integration of digital repositories in staging and response assessment, SR can play a crucial role in clinical decision support systems [9]. SR also fosters opportunities for secondary data capture and multi-center development of registries, ultimately leading to an improved understanding of lung cancer trends and treatment outcomes [2, 8, 9, 26].

### Limitations

While clinical TNM staging is a complex task requiring context-sensitive information, we aimed to assess and isolate the impact of SR on TNM classification compared to FTR. The effect of SR on lesion detection and workflow was not assessed. To account for TNM interdependence, a GLMM was used for statistical analysis. Secondly, the clinical impact of TNM misclassification on clinical management including MDT meetings was not assessed. Thirdly, the proposed 9th edition of the TNM system is anticipated to come into effect in 2025 introducing subclassifications of N2 and M1c classification [30]. This will require updates to the rule-based TNM algorithm. Lastly, the representative cohort of NSCLC patients was small and was assessed in a study setting, which may not be reflective of a standard reading room and clinical conditions.

## Conclusion

This multi-center study yielded a valuable framework for software-assisted SR in NSCLC. Software-assisted SR provided robust data input for semi-automated TNM classification in NSCLC with significantly improved overall performance compared to FTR. A survey among participants revealed that increased workload and lack of digital infrastructure were perceived as the most relevant obstacles to the clinical implementation of SR. The results of this proof-of-concept study suggest a valuable impact of software-assisted SR on TNM correctness in NSCLC staging.

## Data Availability

The questionnaire used in the study is available in the supplements. The relevant data items and entries to develop the software-assisted SR template are available online on the BZKF platform in the German language with relevant RADLEX and SNOMED nomenclature (https://bzkf.de/born-template-lungenkarzinom/).

## Abbreviations

[18F]FDG: 18F-Fluorodeoxyglucose
BORN: Bavarian oncologic radiology network
CI: Confidence interval
ESR: European Society of Radiology
FTR: Free text reporting
GLMM: Generalized linear mixed model
ICC: Intraclass correlation coefficient
MDT: Multidisciplinary team
NSCLC: Non-small cell lung cancer
PACS: Picture archiving and communication system
RSNA: Radiological Society of North America
SR: Structured reporting
